# Assessment of the Risk of Nodal Involvement in Rectal Neuroendocrine Neoplasms: The NOVARA Score, a Multicentre Retrospective Study

**DOI:** 10.3390/jcm11030713

**Published:** 2022-01-28

**Authors:** Angela Dalia Ricci, Sara Pusceddu, Francesco Panzuto, Fabio Gelsomino, Sara Massironi, Claudio Giovanni De Angelis, Roberta Modica, Gianluca Ricco, Martina Torchio, Maria Rinzivillo, Natalie Prinzi, Felice Rizzi, Giuseppe Lamberti, Davide Campana

**Affiliations:** 1 Department of Experimental, Diagnostic and Specialty Medicine, Sant’Orsola-Malpighi University Hospital, ENETS Center of Excellence, 40138 Bologna, Italy; dalia.ricci@gmail.com (A.D.R.); gianluca.ricco@studio.unibo.it (G.R.); davide.campana@unibo.it (D.C.); 2 Division of Medical Oncology, IRCCS Azienda Ospedaliero-Universitaria di Bologna, Via P. Albertoni 15, 40138 Bologna, Italy; 3 Department of Medical Oncology, Fondazione IRCCS Istituto Nazionale Tumori Milano, ENETS Center of Excellence, 20133 Milan, Italy; sara.pusceddu@istitutotumori.mi.it (S.P.); martina.torchio@istitutotumori.mi.it (M.T.); natalie.prinzi@istitutotumori.mi.it (N.P.); 4 Digestive Disease Unit, Sant’Andrea University Hospital, ENETS Center of Excellence of Rome, 00189 Rome, Italy; francesco.panzuto@uniroma1.it (F.P.); mrinzivillo@ospedalesantandrea.it (M.R.); 5 Department of Medical Surgical Sciences and Translational Medicine, Sapienza University of Rome, 00189 Rome, Italy; 6 Division of Oncology, Department of Oncology and Hematology, University Hospital of Modena, 41125 Modena, Italy; fabiogelsomino83@yahoo.it; 7 Division of Gastroenterology, Department of Medicine and Surgery, University of Milano-Bicocca, San Gerardo School of Medicine, 20900 Monza, Italy; sara.massironi@libero.it; 8 Department of Gastroenterology and Digestive Endoscopy, AOU Città della Salute e della Scienza, University of Turin, 10126 Turin, Italy; eusdeang@hotmail.com (C.G.D.A.); rizzifelice91@libero.it (F.R.); 9 Endocrinology, Department of Clinical Medicine and Surgery, ENETS Center of Excellence, University “Federico II” of Naples, 80138 Naples, Italy; robertamodica@libero.it

**Keywords:** rectal neuroendocrine tumor, lymph node metastasis, endoscopic mucosal resection, endoscopic submucosal dissection, low anterior resection

## Abstract

Rectal neuroendocrine tumors (r-NETs) are rare tumors with overall good prognosis after complete resection. However, there is no consensus on the extension of lymphadenectomy or regarding contraindications to extensive resection. In this study, we aim to identify predictive factors that correlate with nodal metastasis in patients affected by G1–G2 r-NETs. A retrospective analysis of G1–G2 r-NETs patients from eight tertiary Italian centers was performed. From January 1990 to January 2020, 210 patients were considered and 199 were included in the analysis. The data for nodal status were available for 159 cases. The nodal involvement rate was 9%. A receiver operating characteristic (ROC) curve analysis was performed to identify the diameter (>11.5 mm) and Ki-67 (3.5%), respectively, as cutoff values to predict nodal involvement. In a multivariate analysis, diameter > 11.5 mm and vascular infiltration were independently correlated with nodal involvement. A risk scoring system was constructed using these two predictive factors. Tumor size and vascular invasion are predictors of nodal involvement. In addition, tumor size > 11.5 mm is used as a driving parameter of better-tailored treatment during pre-operative assessment. Data from prospective studies are needed to validate these results and to guide decision-making in r-NETs patients in clinical practice.

## 1. Introduction

Rectal neuroendocrine tumors (r-NETs) represent a heterogeneous group of rare malignancies that account for up to 13.7% of all neuroendocrine tumors (NETs) [1]. According to the Surveillance, Epidemiology, and End Results registry database of the National Cancer Institute, the age-adjusted incidence of r-NETs has increased about sixfold over the last 40 years, probably due to the increased use of endoscopic procedures for colorectal cancer screening [2]. R-NETs typically appear as single smooth yellowish polypoid lesions that originate from deeper layers of the mucosa and protrude from the mucosal surface into the lumen of the rectum without surface distortion [3,4]. The 2016 European Neuroendocrine Tumour Society (ENETS) guidelines recommend different surgical approaches of R-NETs, including endoscopic mucosal resection (EMR), endoscopic submucosal dissection (ESD), transanal endoscopic microsurgery (TEMS), and low anterior resection (LAR) depending on the tumor size, endoscopic ultrasound staging (T and N), and World Health Organization (WHO) grading (G1/2 or G3) [5,6]. Nevertheless, there is no consensus on the extension of lymphadenectomy or contraindications to extensive resection.

Rectal lesions less than 10 mm in size typically show an indolent course, with the nodal involvement incidence ranging from 1% to 10%, a high rate of curative resection, and 5-year survival of 98 to 100%. Conversely, in the case of r-NETs 10 mm to 20 mm and larger than 20 mm in size, the reported incidence of nodal involvement increases to 30% and 60%, respectively, with a worse prognosis [7,8,9]. Previous studies have shown that a tumor size of >10 mm or >20 mm, stage, depth of submucosal invasion, lymphovascular invasion (LVI), or tumor grade 3 (G3) are important predictors of lymph node metastases, but the risk factors for nodal involvement have not been clearly elucidated [3,10,11,12,13,14].

This study aimed at identifying potential clinical and histopathological risk factors for lymph node metastases and to construct a risk stratification score relevant for determining the proper treatment option in G1–G2 r-NETs.

## 2. Materials and Methods

### 2.1. Study Design and Participants

This was a retrospective analysis of a multicentric prospective database of 210 consecutive patients affected by r-NETs referred to 7 tertiary Italian centers from January 1990 to January 2020. The study was approved by the local Institutional Review Board (Comitato Etico Indipendente, S.Orsola-Malpighi Hospital, Bologna, Italy) and was conducted in accordance with the principles of the Declaration of Helsinki (revision of Edinburgh, 2000). The primary endpoint of this study was the identification of predictive factors related to the presence of nodal involvement in patients with r-NETs.

All consecutive patients undergoing endoscopic or surgical resection of r-NETs at 7 tertiary Italian centers during the study period were included and provided informed consent at the time of surgery for anonymous review of their data for research purposes. Patients with neuroendocrine carcinoma (NEC) G3 (according to WHO 2010 classification), mixed adenoneuroendocrine carcinoma (MANEC), or no evidence of r-NETs on pathology revision were excluded from the analysis. Nodal involvement was defined on the basis of pathology report in surgically resected patients or of unequivocal imaging finding (magnetic resonance, endoscopic ultrasonography, or PET with Ga-DOTA-peptide). Indeed, endoscopic ultrasonography in addition to MRI and PET/CT is an accurate tool to capture nodal metastases even if pathologic nodal status is not confirmed. Data about nodal involvement were not available if patients did not undergo surgical resection or had no proper imaging.

### 2.2. Data Collection

All data were prospectively collected at the center where surgery was performed for every patient. A single computerized data sheet was created and patient demographics, clinical presentation, surgical, and pathological characteristics were retrospectively analyzed. Data collected included: gender, age, onset of symptoms, endoscopic features (presence of ulceration, presence of depressed lesion, or multiple lesions), type of endoscopy resection, and/or of surgical procedures performed. The gathering of data from 7 tertiary Italian centers provides a picture that reflects the risk profile for lymph node metastases in routine hospital care.

### 2.3. Pathology Assessment

Pathological features, such as tumor size, localization site according to the European Society for Medical Oncology (ESMO) guidelines definition for rectal carcinoma (<5 cm beginning at the anal verge as low, 5–10 cm as mid, and 10–15 cm as high rectal cancer), lymphovascular and perineural invasion, Ki-67, WHO 2010 classification (used at the time of histopathological exams), and the ENETS grading system, were listed [15,16,17]. Ki-67 values are expressed as the percentage of positively marking malignant cells using the anti-human Ki-67 monoclonal antibody MIB1. The margin clearance was not available since the review of tissue samples was not performed for the retrospective study design. All specimens were examined by a NET expert pathologist at each center.

### 2.4. Statistical Analysis

Categorical variables are expressed as numbers and percentages and compared using the chi-squared test or Fisher’s exact test when appropriate. Continuous variables are expressed as medians and interquartile range (IQR, 25th to 75th percentiles) and compared using Mann–Whitney U test. Receiver-operating characteristic (ROC) curve was built to identify the best cutoff value for the prediction of nodal involvement according to the size of the tumor and Ki67 value. Analysis of the predictive factors of nodal disease was carried out by univariate and multivariate analysis using logistic regression. Predictive factors were expressed as odds ratio (OR) and 95% confidence interval (95% CI). A value of *p* < 0.05 was considered statistically significant. Statistical analyses were performed using SPSS Statistics v. 22 (IBM).

## 3. Results

### 3.1. Study Population

**Figure 1:**
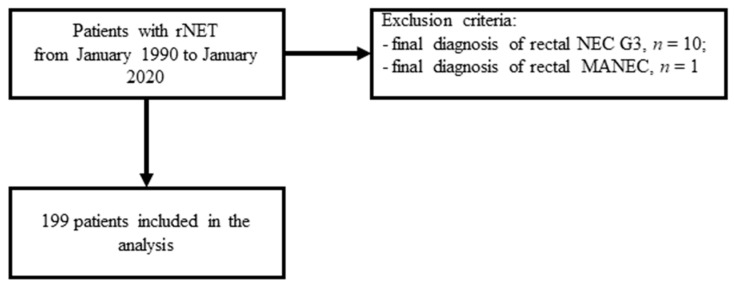
Study flow chart. rNET: rectal neuroendocrine tumor; NEC: neuroendocrine carcinoma; MANEC: mixed adenoneuroendocrine carcinomas.

Of the 210 patients considered for the analysis, eleven patients were excluded, ten because they were affected by rectal NEC and one for being affected by MANEC. The remaining 199 patients met the inclusion criteria and were included in the analysis. The selection process is shown in Figure 1.

The median age at diagnosis was 55 (IQR 46–62), 47.2% of patients were female (*n* = 90), and 52.8% were male (*n* = 105). All the patients underwent endoscopic or surgical resection that was performed by polypectomy (*n* = 125, 62.8%), EMR (*n* = 22, 11.1%), ESD (*n* = 14, 7.0%), TEMS (*n* = 8, 4.0%), or LAR (*n* = 12, 6.0%). Type of resection was not specified in 9.1% of the cases (*n* = 18). Sixty-six (33.1%) patients were diagnosed following symptomatic presentation, including diarrhea, rectal bleeding, anemia, abdominal pain, and tenesmus. According to ENETS-TNM staging, 181 patients (91.0%) presented with clinical stage I-IIIA, while 18 patients (9.0%) presented with clinical IIIB-IV stage [17]. In 111 cases, (55.8%) tumors were located in the high/medium rectum, in 72 (36.2%) in the low rectum. The median tumor size was 6 mm (IQR 3–9.25 mm). The median Ki-67% value was 1% (IQR 1–2%). According to WHO 2010 classification, 147 patients (73.9%) had G1 NET and 41 patients (20.6%) had G2 NET; data were not reported in 11 (5.5%) cases. Vascular, perineural, and lymphatic invasion were observed in 16 cases (8%), 11 cases (5.5%), and 11 cases (5.5%), respectively. Among the entire cohort, 18 of the 199 patients had positive lymph nodes (9%); data were not available for 40 patients (20.1%). The baseline patient and tumor characteristics are summarized in Table 1.

### 3.2. ROC Curves

**Figure 2:**
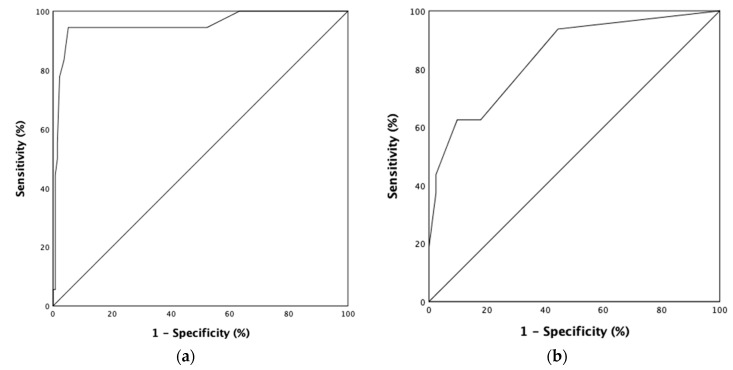
ROC curve for the best cutoff value of tumor size and Ki-67 predicting nodal involvement. (**a**) Area under ROC curve (AUROC) for tumor size: 0.953; 95% confidence interval: 0.891–1.000; standard error: 0.032; cutoff value: 11.5 mm; (**b**) area under ROC curve (AUROC) for Ki-67: 0.843; 95% confidence interval: 0.737–0.949; standard error: 0.054; cutoff value: 3.5%.

Two ROC curves of the tumor size and Ki-67 were used to determine the best cutoff values predicting nodal involvement. The best tumor size cutoff value for nodal involvement was 11.5 mm (area under the curve standard error, 0.747 ± 0.032; Figure 2a). In the cohort, twenty-six (13.1%) patients presented with r-NETs > 11.5 mm, and, among them, 16 (61.5%) patients had nodal involvement. On the other hand, the best point for Ki-67 predicting nodal involvement was >3.5% (area under the curve standard error, 0.843 ± 0.054) (Figure 2b). Twenty-six (13.1%) patients had Ki-67 > 3.5%, and, among them, 7 (30%) patients had nodal involvement.

### 3.3. Univariate Analysis, Multivariate Analysis, and Prognostic Score Development

Firstly, we performed univariate and multivariate analyses of the clinical characteristics related to nodal involvement. Tumor size > 11.5 mm (OR:333.3; *p* < 0.001), ulceration (OR:16.1; *p* < 0.001), and depression (OR:10.7; *p* < 0.001) were significantly associated with nodal involvement at univariate analysis. On multivariate analysis, tumor size (OR:575.2; *p* < 0.001) was the only variable independently related to nodal involvement (Supplementary Table S1). Then, univariate and multivariate analyses of histopathological factors related to nodal involvement were carried out. Ki-67 > 3.5% (OR:19.0; *p* < 0.001), muscle layer invasion (OR:61; *p* < 0.001), vascular invasion (OR:59.5; *p* < 0.001), perineural invasion (OR:244.0; *p* < 0.001), and lymphatic invasion (OR:246.0; *p* < 0.001) were related with nodal involvement. Multivariate analysis (Supplementary Table S2) confirmed that Ki-67 > 3.5% (OR:95.4; *p* < 0.002), muscle layer invasion (OR:39.1; *p* < 0.005), and vascular invasion (OR:61.6; *p* < 0.005) were independently associated with the presence of nodal involvement. Finally, a multivariate analysis on the combined clinical and histopathological factors related to nodal involvement was performed (Table 2). Tumor size > 11.5 mm (OR:54.9; *p* < 0.002) and vascular invasion (OR:51.3; *p* < 0.007) retained their association with nodal involvement. Muscle layer invasion and Ki-67 > 3.5% were not related to nodal involvement at multivariate analysis.

**Figure 3:**
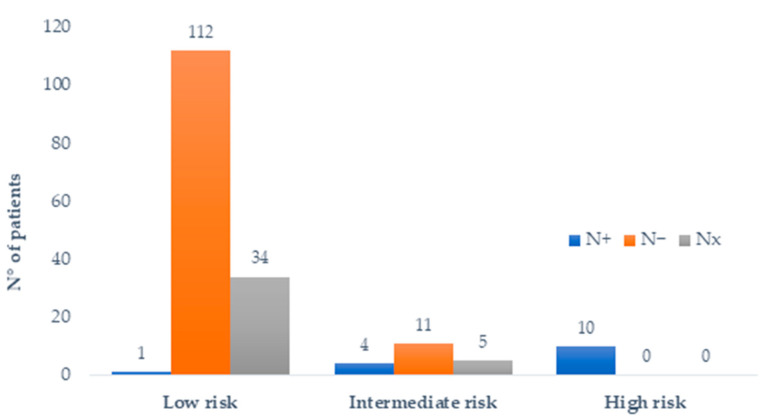
Distribution of patients and nodal status according to NOVARA score. Nx: unknown nodal status; N−: absence of lymph node metastases; N+: presence of lymph node metastases.

On this basis, we created a predictive model of nodal involvement by combining the two clinicopathological variables within the NOVARA score (assessment of the risk of nodal involvement in rectal neuroendocrine neoplasms) and by assigning weight 1 to each of the following variables: tumor size > 11.5 mm and presence of vascular invasion. Accordingly, the patients were stratified into three different risk groups as follows: low-risk group (zero predictive factors), intermediate-risk group (one predictive factor), and high-risk group (two predictive factors). Among the patients with both tumor size and LVI status available, 147 (83%) of the patients were categorized as low-risk, 20 (11%) patients as intermediate-risk, and 10 (6%) as high-risk. The data regarding tumor size and/or vascular invasion were not reported in 22 (11%) of the patients. Of the 147 low-risk patients, the data on regional lymph node status were available in 113 cases and nodal involvement was found in one case (0.9%). Of the 20 intermediate-risk patients, lymph node metastases were noted in four of the fifteen patients with known lymph node status (26.7%). Of the 10 high-risk patients, all the patients had known nodal status and all the patients presented with nodal involvement (100%; Figure 3).

## 4. Discussion

We evaluated the clinicopathological risk factors related to nodal involvement in a large cohort of newly diagnosed patients with G1–G2 r-NETs. Furthermore, we provided initial evidence of a predictive score that takes into account tumor size and vascular invasion.

The incidence of r-NETs has been increasing in recent decades and, despite the overall good prognosis, the long-term prognosis of r-NETs is comparable to that of colorectal cancer in the case of nodal involvement [13,18,19]. Thus, a risk stratification-based approach could suggest the appropriate surgical or endoscopic management in this setting.

Previous studies reported a correlation between primary tumor size and the likelihood of lymph node metastases in r-NETs [3,10,11,20,21,22,23,24,25,26,27,28]. Therefore, the National Comprehensive Cancer Network (NCCN) guidelines and the latest ENETS guidelines recognize the identification of tumor size as a major parameter to determine the patient prognosis and therapy options [5,29]. According to ENETS guidelines, any decision regarding the therapeutic approach is based on the assessment of tumor size, muscle layer invasion, grading, and presence of regional or distant metastases. Tumors that are smaller than 10 mm and well-differentiated should be completely removed endoscopically, whereas r-NETs larger than 20 mm, which are more likely to invade muscularis propria and to have malignant potential, should be considered for surgical resection [5,9]. On the other hand, there is still no consensus regarding r-NETs of intermediate size (10–19 mm), where an accurate tumor assessment by endoscopy and endoanal ultrasound should guide towards an endoscopic, transanal, or surgical approach [30,31].

We showed that tumor size greater than 11.5 mm and vascular invasion were independent risk factors for lymph node metastases. Nevertheless, despite what is reported in the current ENETS guidelines, our investigation lowered the dimensional cutoff for clinical decisions from 20 mm to 11.5 mm in line with the results from the latest retrospective analyses regarding r-NETs [13,32,33,34,35]. Similar to our findings, two large retrospective studies based on national registries published in 2019 and a retrospective report from the French group of endocrine tumors (GTE) confirmed that tumor size larger than 10 mm was related to nodal involvement in non-metastatic r-NETs, along with other predictive factors, such as tumor grade and presence of muscular and lymphovascular invasion [33,34,35]. Another retrospective registry-based study by Concors et al. found that the cutoff value of 11.5 mm was also able to predict the risk of distant metastases in well-differentiated and moderately differentiated r-NETs, suggesting a possible role for radical surgical resection in these cases [32]. With regard to vascular invasion, defined by the presence of tumor cells in blood vessels, our findings were in agreement with the available literature, suggesting its predictive role of nodal involvement [36,37]. The prevalence of LVI in small r-NETs was 21.8% according to a recent systematic review and meta-analysis by Kang et al., and, when separately analyzed, the vascular invasion had a stronger impact on lymph node metastasis than the lymphatic invasion [38].

Moreover, we found a Ki-67 > 3.5% as the optimal cut-point value for the risk of nodal metastases. However, Ki67 did not retain its association with the risk of nodal involvement upon multivariate analysis. Nonetheless, of the 199 consecutive patients considered for the analysis, nodal involvement was found in 18 (9%) of the cases and, among these, 10 (55.6%) patients presented with tumor size larger than 11.5 mm and vascular invasion. The combination of these two single parameters in the NOVARA risk prediction score, of which tumor size can be assessed preoperatively, has led to differentiate three different categories with a distinct risk of nodal involvement that could allow discussion for better-tailored treatment and a dedicated surveillance program. Thus, the NOVARA score can identify patients with a low risk of nodal involvement that are likely to have an excellent prognosis and benefit from endoscopic resection, and patients with intermediate to high risk that should be considered for surgical resection and/or close monitoring.

The retrospective design of our study, along with the use of a large dataset with certain missing data, are two limitations to be acknowledged. Particularly, the main limitation is the lack of long-term follow-up data, which precludes the possibility to analyze the impact of lymph node metastases on survival outcomes. Moreover, since nodal pathology or imaging was not performed in all the patients as per standard clinical practice, occult metastases might have been underestimated in some patients, and this could have led to a selection bias. Nevertheless, to our knowledge, this is the first Italian multicentric study and one of the few non-registry-based studies that assessed the predictors of nodal involvement in a wide cohort of patients with G1–G2 r-NETs. Additionally, given the paucity of dedicated high-level evidence, our study developed a scoring system for risk stratification that can be incorporated in clinical practice and help guide discussions with patients regarding their risk of lymph node metastases.

## 5. Conclusions

We covered one of the largest multicenter studies conducted on this topic so far. According to our results, tumor size and vascular invasion predicted nodal involvement and were incorporated in the NOVARA predictive score, according to which patients presenting both factors had a higher risk of nodal involvement at diagnosis and should thus be considered for radical surgical resection. In addition, our findings suggest that tumor size > 11.5 mm is a fundamental variable guiding the most appropriate surgical approach during pre-operative assessment. In our view, well-designed, prospective clinical trials are required to validate these results and to guide decision-making in r-NETs patients in everyday clinical practice worldwide.

## Figures and Tables

**Table 1:** Baseline characteristics of the study population.

Characteristics	Patients (*n* = 199)
Demographic	
Gender, male No. (%)	105 (52.8%)
Gender, female No. (%)	90 (47.2%)
Age, median (IQR), years	55 (46–62)
Presence of symptoms	
Yes (%)	66 (33.1%)
No (%)	121 (60.8%)
Not available (%)	12 (6.1%)
TNM staging	
Stage I-IIA (%)	181 (91.0%)
Stage IIIB-IV (%)	21 (9.0%)
Site	
High/medium rectum (%)	111 (55.8%)
Low rectum (%)	72 (36.2%)
Not available (%)	16 (8.0%)
Resection	
Polypectomy (%)	125 (62.8%)
EMR (%)	22 (11.1%)
ESD (%)	14 (7.0%)
TEMS (%)	8 (4.0%)
LAR (%)	12 (6.0%)
Not available (%)	18 (9.1%)
Pathological features	
Size, median (IQR), mm	6 (3.0–9.25)
Ulceration (%)	18 (9.0%)
Depression (%)	16 (8.0%)
Synchronous lesions (%)	13 (6.5%)
Vascular invasion (%)	16 (8.0%)
Perineural invasion (%)	11 (5.5%)
Lymphatic invasion (%)	11 (5.5%)
WHO 2010 Classification	
NET G1 (%)	147 (73.9%)
NET G2 (%)	41 (20.6%)
Not available (%)	11 (5.5%)
Ki-67, median (%)	1% (1–2%)
Nodal involvement	
Yes (%)	18 (9.0%)
No (%)	141 (70.9%)
Not available (%)	40 (20.1%)

EMR, endoscopic mucosal resection; ESD, endoscopic submucosal dissection; TEMS, transanal endoscopic microsurgery; LAR, low anterior resection.

**Table 2:** Multivariate analysis of clinical and histopathological factors predicting nodal involvement in G1–G2 r-NEN.

Characteristics	Multivariate Analysis		
	OR	IC 95%	*p*
Size > 11.5 mm	54.9	4.2–711.0	0.002
Ki67 > 3.5%	-	-	ns
Muscle layer invasion	-	-	ns
Vascular invasion	51.3	2.9–906.7	0.007

ns: not significant.

## Data Availability

The data presented in this study are available on reasonable request from the corresponding author. The data are not publicly available due to privacy reasons.

## Supplementary Materials

The following supporting information can be downloaded at: https://www.mdpi.com/article/10.3390/jcm11030713/s1, Table S1: Univariate and multivariate analysis of clinical factors predicting nodal involvement in G1-G2 r-NEN. Ns: not significant; Table S2: Univariate and multivariate analysis of anatomopathological factors predicting nodal involvement in G1-G2 r-NEN. Ns: not significant.
