# Supplementary material for: Assessment of the Risk of Nodal Involvement in Rectal Neuroendocrine Neoplasms: The NOVARA Score, a Multicentre Retrospective Study
Source: J Clin Med. 2022 Jan 28;11(3):713. doi: 10.3390/jcm11030713 (PMC8836953; doi:10.3390/jcm11030713)
Supplement: Supplementary file 1 [file jcm-11-00713-s001.zip › jcm-1556614-supplementary.pdf]

Supplementary Materials

Supplementary Table S1. Univariate and multivariate analysis of clinical factors predicting nodal involvement in G1-G2 r-NEN. Ns: not significant.

Characteristic	Univariate Analysis			Multivariate Analysis		
	**OR**	**IC95%**	**p**	OR	IC 95%	p
**Male (sex)**	2.0	0.7–5.6	0.198			ns
**Age**	1.0	1.0–1.0	0.964			ns
**Low rectum**	1.4	0.5–3.9	0.489	14.9	0.9–241.9	0.057
**Symptom onset**	7.5	2.3–24.2	0.001	6.5	0.6–68.5	0.121
**Synchronous lesions**	0.7	0.1–6.0	0.768			ns
**Size > 11.5mm**	333.3	36.3–2704.2	<0.001	575.2	25.7–12883.6	<0.001
**Ulceration**	16.1	4.8–54.6	<0.001			ns
**Depression**	10.7	3.1–37.7	<0.001			ns

Supplementary Table S2. Univariate and multivariate analysis of anatomopathological factors predicting nodal involvement in G1-G2 r-NEN. Ns: not significant.

Characteristic	Univariate Analysis			Multivariate Analysis		
	**OR**	**IC95%**	**p**	OR	IC95%	p
**Ki67 > 3.5%**	19.0	5.7–63.1	<0.001	95.4	5.0–1811.4	0.002
**Muscle layer invasion**	61.0	14.3–260.9	<0.001	39.1	3.0–508.2	0.005
**Vascular invasion**	59.5	13.8–257.4	<0.001	61.8	3.4–1112.9	0.005
**Perineural invasion**	244.0	25.9–22956.0	<0.001			ns
**Lymphatic invasion**	246.0	26.1–2314.6	<0.001			ns
